# Perspective of 3D culture in medicine: transforming disease research and therapeutic applications

**DOI:** 10.3389/fbioe.2024.1491669

**Published:** 2024-12-19

**Authors:** Chan Hum Park, Jung Ho Park, Yong Joon Suh

**Affiliations:** ^1^ Nano-Bio Regenerative Medical Institute, College of Medicine, Hallym University, Chuncheon, Republic of Korea; ^2^ Departments of Otorhinolaryngology-Head and Neck Surgery, Chuncheon Sacred Heart Hospital, School of Medicine, Hallym University, Chuncheon, Republic of Korea; ^3^ Department of Breast and Endocrine Surgery, Hallym University Sacred Heart Hospital, Anyang, Republic of Korea

**Keywords:** 3D culture, stem cell, scaffold, biomaterial, bioprinting, medicine

## Abstract

3D cell culture is gaining momentum in medicine due to its ability to mimic real tissues (*in vivo*) and provide more accurate biological data compared to traditional methods. This review explores the current state of 3D cell culture in medicine and discusses future directions, including the need for standardization and simpler protocols to facilitate wider use in research.

**Purpose:**

3D cell culture develops life sciences by mimicking the natural cellular environment. Cells in 3D cultures grow in three dimensions and interact with a matrix, fostering realistic cell behavior and interactions. This enhanced model offers significant advantages for diverse research areas.

**Methods:**

By mimicking the cellular organization and functionalities found in human tissues, 3D cultures provide superior platforms for studying complex diseases like cancer and neurodegenerative disorders. This enables researchers to gain deeper insights into disease progression and identify promising therapeutic targets with greater accuracy. 3D cultures also play a crucial role in drug discovery by allowing researchers to effectively assess potential drugs’ safety and efficacy.

**Results:**

3D cell culture’s impact goes beyond disease research. It holds promise for tissue engineering. By replicating the natural tissue environment and providing a scaffold for cell growth, 3D cultures pave the way for regenerating damaged tissues, offering hope for treating burns, organ failure, and musculoskeletal injuries. Additionally, 3D cultures contribute to personalized medicine. Researchers can use patient-derived cells to create personalized disease models and identify the most effective treatment for each individual.

**Conclusion:**

With ongoing advancements in cell imaging techniques, the development of novel biocompatible scaffolds and bioreactor systems, and a deeper understanding of cellular behavior within 3D environments, 3D cell culture technology stands poised to revolutionize various aspects of healthcare and scientific discovery.

## Introduction

Three-dimensional (3D) cell culture models aim to recreate natural environments outside the body, allowing cells to grow and interact in three dimensions (Haycock, 2011; Ravi et al., 2015; Gu et al., 2020; Yoon, 2023). By culturing various cell types within a 3D extracellular matrix, nutrients, oxygen, and drugs can efficiently reach cells, closely mimicking physiological conditions (Lee et al., 2019). Traditional two-dimensional (2D) cell culture systems, while convenient and cost-effective, lack the ability to accurately replicate the complex architecture and microenvironment found in living tissues (Duval et al., 2017). This limitation can lead to misleading results, particularly regarding the response of cancer cells to anticancer agents, as 2D cultures fail to mimic the true 3D tumor microenvironment (Gao et al., 2017; Brancato et al., 2020).

The emergence of 3D cell culture systems presents promising solutions to bridge the gap between laboratory cell cultures and *in vivo* conditions (Alghuwainem et al., 2019; Diaz-Rodriguez et al., 2019; Mofazzal Jahromi et al., 2019; Lin et al., 2020; Terrell et al., 2020). These systems offer higher physiological relevance, closely resembling cell behavior within the body, and provide new avenues for cell-based research and clinical trials (van Duinen et al., 2015; Bertucci et al., 2019; Calejo et al., 2019; Ham et al., 2019; Thelu et al., 2020). The intricacy inherent in 3D systems presents multifaceted challenges, notably in the selection of scaffold materials and cell types (Ashok et al., 2020). Researchers are confronted with the delicate decision between natural and synthetic scaffold materials, as well as the nuanced choice between utilizing autologous or adult-derived stem cells. Moreover, the meticulous fabrication of nanoscale scaffolds or the creation of microscale structures with precise architectures to support cell growth requires meticulous deliberation.

The dynamic nature of 3D cultures, moreover, introduces complexities in maintaining optimal culture environments over prolonged periods (Fernandes et al., 2020). Unlike static 2D cultures, 3D systems demand sophisticated strategies to facilitate nutrient diffusion, oxygenation, and waste removal throughout the entire structure. Innovations such as perfusion bioreactors and microfluidic systems have been devised to tackle these challenges, offering enhanced control over culture conditions, and ensuring the sustained viability of 3D constructs.

The integration of diverse cell types within 3D culture systems introduces yet another layer of intricacy (Ryu et al., 2019). Replicating the heterogeneous composition of tissues and organs *in vivo* often necessitates the co-cultivation of disparate cell populations to accurately simulate physiological interactions. This interdisciplinary endeavor mandates seamless collaboration among cell biologists, materials scientists, engineers, and clinicians to meticulously design and optimize 3D culture platforms tailored for specific applications, encompassing surgical research and regenerative medicine.

This review explores the current state of 3D culture in medicine and discusses future directions, such as standardization and simpler protocols, to facilitate broader adoption in surgical research. Addressing these challenges will be crucial for realizing the possibilities of 3D cell culture in medical applications and beyond.

### Environments

**FIGURE 1:**
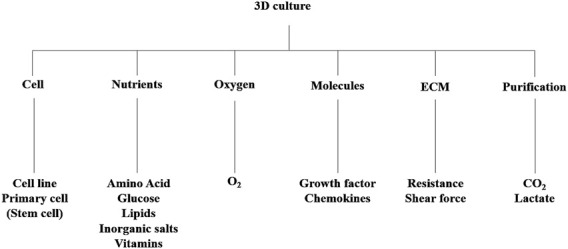
The components for 3D cell culture are shown here. ECM, extracellular matrix.

Essential cultural platforms in biomedical research have been investigated particularly for drug discovery and anticancer investigations (Kim et al., 2020). The rising demand for advanced 3D cell culture models has intensified the need for sophisticated 3D cultivation techniques, from laboratory setups to industrial-scale production (Brancato et al., 2020; Wan et al., 2020). These bioreactors boast precise control systems, ensuring reproducible spheroid formation, a vital aspect for studying cellular behavior and drug responses. Beyond conventional applications, bioreactors play a crucial role in facilitating dynamic cell interactions and responses (Ferreira et al., 2018). They offer precise control over environmental conditions, including temperature, pH, and nutrient supply, ensuring optimal cell growth and function (Figure 1). Additionally, advancements in bioreactor design enable the integration of various monitoring and control systems for enhanced functionality.

Utilizing established 3D culture models, researchers have made significant strides in understanding cancer progression mechanisms (Dumont et al., 2019; Zhang et al., 2020). These models provide insights into morphological and cellular changes associated with disease progression, offering valuable platforms for drug screening and efficacy testing. Real-time monitoring of 3D cultures allows for the assessment of dynamic cellular behaviors, such as proliferation, migration, and interaction (De Leon et al., 2020). This capability is instrumental in studying complex phenomena like angiogenesis and tumor invasion, providing insights into disease mechanisms and potential therapeutic interventions.

Progression models enable the evaluation of treatment responses and the identification of novel therapeutic targets, contributing to advancements in precision medicine and personalized therapies (Xia et al., 2019). Furthermore, these models aid in deciphering intricate cellular signaling pathways involved in disease progression, paving the way for targeted interventions. Accurate measurement of structural changes in 3D cultures requires precise labeling techniques to track cell proliferation and migration over time (Zhang et al., 2019). Various labeling methods, such as fluorescent proteins and dyes, enable researchers to monitor multiple cell types simultaneously, facilitating comprehensive analyses of complex cellular interactions (Linsley et al., 2019).

### 3D constructs

Compared to traditional 2D cell cultures, 3D scaffolds provide a more realistic environment for cellular growth and interaction (Gupta et al., 2019). Crafted from diverse materials, these structures offer controlled porosity, permeability, surface chemistry, and mechanical properties, closely resembling the native extracellular matrix (ECM) that supports cellular organization in tissues (Bayir et al., 2019; Huang et al., 2020). This porous network not only supports cellular growth but also facilitates nutrient and oxygen diffusion, essential for maintaining cell viability and function (Terrell et al., 2020).

Fabricating these scaffolds entails various techniques, each tailored to achieve specific structural and mechanical properties (Ashok et al., 2020). Freeze-drying, for instance, utilizes sublimation to create a porous scaffold from a frozen solution, while electrospinning produces fibrous scaffolds through the application of electrostatic forces (Fan et al., 2019). The applications of 3D scaffolds are vast, spanning both tissue engineering and cell culture research. In tissue engineering, these scaffolds serve as a scaffold for the regeneration of damaged organs and tissues. By seeding scaffolds with appropriate cells and growth factors, researchers aim to facilitate tissue repair and functional restoration.

Beyond tissue regeneration, 3D scaffolds play a pivotal role in advancing our understanding of disease mechanisms and cell biology. By providing a more physiologically relevant environment, these scaffolds enable researchers to study cell-cell interactions, drug responses, and disease progression with greater accuracy. 3D scaffolds stand as indispensable tools in tissue engineering and cell culture for studying cellular behavior and advancing regenerative medicine.

### Cells

Stem cells, particularly pluripotent stem cells (PSCs), have garnered immense interest due to their ability to generate various cell types and their potential in regenerative medicine (Costamagna et al., 2019; Turco and Moffett, 2019). They offer promising avenues for medical innovation, including drug discovery, cell therapy, and tissue regeneration (Artero Castro et al., 2019; Srivastava and Kilian, 2019). Utilizing advanced 3D cell platforms, researchers have made significant strides in understanding cell signaling and tissue development, particularly in fields.

Culturing cells in 3D models has emerged as a vital tool, providing results that closely mimic natural conditions and aiding in the translation of research findings into clinical applications (Alagarsamy et al., 2019; Balak et al., 2019; Chen and Schoen, 2019; Gibbs et al., 2019; Gopalakrishnan, 2019; Meivar-Levy and Ferber, 2019; Roberts et al., 2019; Nguyen et al., 2020; Raimondi et al., 2020). These models offer a more realistic representation of cellular behavior, ensuring better predictability when moving from the laboratory to real-world settings. Autologous, allogeneic, and xenogeneic cells are employed, with autologous cells preferred to mitigate the risk of rejection (Collins et al., 2020).

Adipose-derived stem cells (ASCs) have emerged as a significant player in regenerative medicine, offering several advantages over other sources such as bone marrow-derived mesenchymal stem cells (MSCs) (Ryu et al., 2019). ASCs, found within adipose tissue, boast higher yields and greater resistance to senescence, making them an attractive option for therapeutic applications (Seo et al., 2019). However, challenges persist in standardizing isolation methods and understanding their precise characteristics. The unique characteristics of ASCs vary depending on the tissue type and isolation method employed. Techniques like power-assisted liposuction have shown promising results in yielding high-quality ASCs, underscoring the importance of optimization in isolation protocols. Research continues to elucidate ASC behavior and identify specific markers to distinguish them from other cell types accurately.

Despite the progress made in harnessing the potential of stem cells and ASCs, several hurdles remain to be addressed. Standardization of isolation methods, precise differentiation instructions, and clarification of cell characteristics are crucial areas requiring further investigation. Nevertheless, the ongoing research holds significant promise for revolutionizing medical treatments and offering hope to patients with various degenerative diseases and injuries (Sthijns et al., 2019).

### Medical applications

**FIGURE 2:**
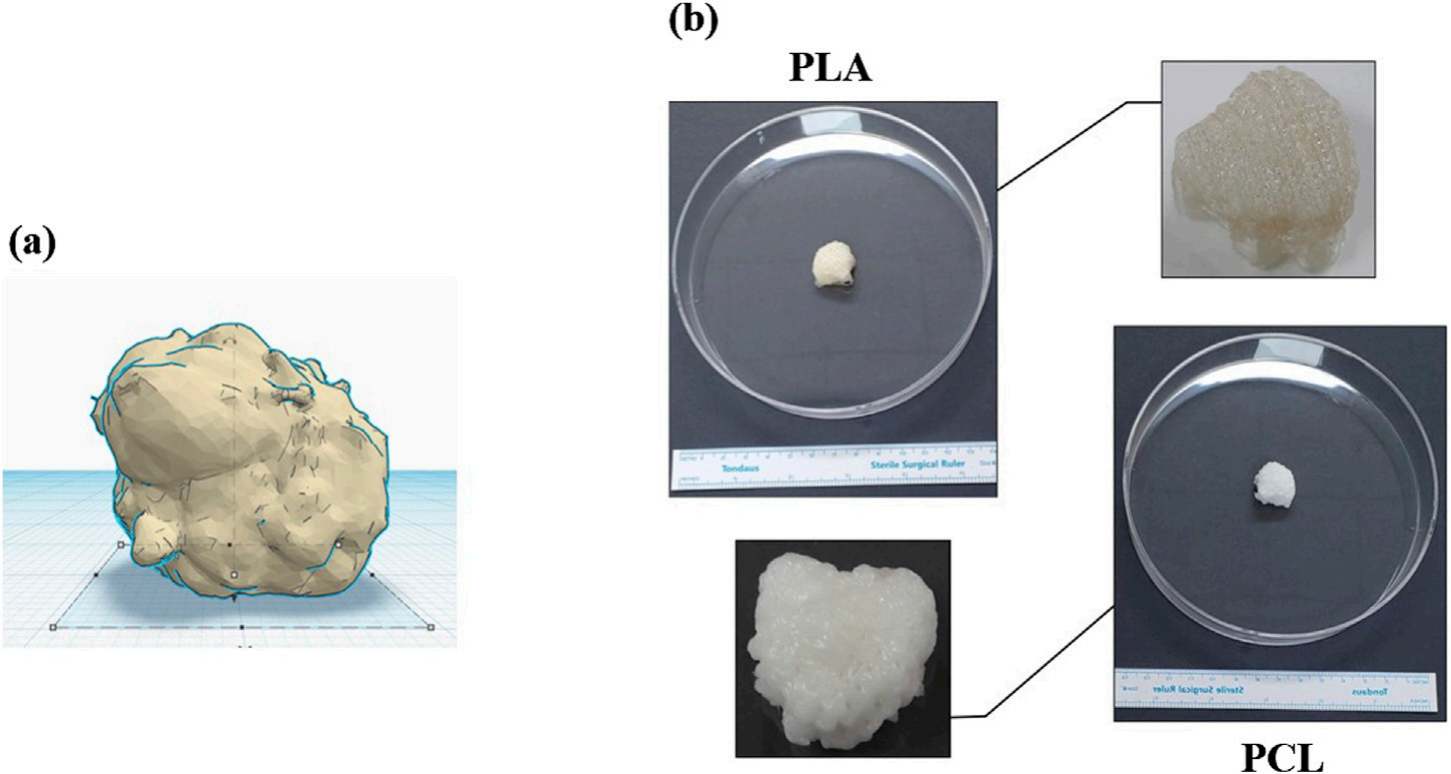
The pictures show: **(A)** a defect after oncologic surgery, and **(B)** a 3D bioprinted scaffold (PCL/PLA, MediFab Inc.) for reconstruction. PLA, polylactic acid; PCL, polycaprolactone.

Transitioning from traditional 2D cell culture systems to more advanced 3D approaches represents a significant step towards understanding cellular behavior within a physiologically relevant context (Ndyabawe and Kisaalita, 2019; Jensen and Teng, 2020). 3D culture methods offer a more accurate representation of *in vivo* conditions, bridging the gap between conventional cell culture systems and the intricate physiology of living organisms (Figure 2). This advancement is particularly crucial in the medical research, where cellular interactions and the microenvironment play pivotal roles in tumorigenesis (Chaicharoenaudomrung et al., 2019).

These benefits in tissue engineering and regenerative medicine enable 3D culture to be considered for various clinical applications (Table 1). The previous studies showed the development of 3D bioprinted skin tissue, adipose microtissues, and bone scaffolds, demonstrating substantial improvements in cell viability, differentiation, and functionality (Gholipourmalekabadi et al., 2018; Yan et al., 2019; Yang et al., 2021; Li et al., 2024). Studies on esophageal, gastric, and intestinal organoids provide insights into stem cell potential and disease modeling, revealing the intricate processes of tissue development and regeneration (Spence et al., 2011; DeWard et al., 2014; McCracken et al., 2014). The generation of vascularized liver buds, lung organoids, and pancreatic organoids underscores the progress in creating functional human tissues for therapeutic applications, offering promising avenues for treating related diseases (Takebe et al., 2013; Dye et al., 2015; Boj et al., 2016). The researches on heart defect, prostate cancer, optic cup formation, inner ear sensory tissue, and lingual epithelium organoids emphasize the importance of organoid systems in understanding disease and developing personalized treatments. They suggested innovative approaches in heart patch, musculoskeletal tissue engineering, cartilage repair, and ophthalmopathy treatment, focusing on the potential of stem cell-derived tissues and hybrid scaffolds in regenerative medicine (Eiraku et al., 2011; Hisha et al., 2013; Gao et al., 2014; Koehler and Hashino, 2014; Fischer et al., 2023; Soucy et al., 2025). Some studies explore the possible usage of β -cell generated from human pluripotent stem cells to cure diabetes mellitus, the potential of salivary gland stem cell therapy for treating xerostomia, and the development of bioprinted human skin substitutes (Lee et al., 2014; Nanduri et al., 2014; Millman et al., 2016; Baltazar et al., 2023). The researches on liver organoids derived from primary human hepatocytes and the creation of hybrid scaffolds for musculoskeletal tissue demonstrate the ongoing efforts to enhance the complexity and functionality of engineered tissues (Salas-Silva et al., 2023; Enbergs et al., 2024).

**TABLE 1:** The clinical application of 3D cell culture in medical areas.

Researcher (year)	Cell	Condition	Application	References
Gholipourmalekabadi M. (2018)	ASC	Burn	Skin	Gholipourmalekabadi et al. (2018)
Yang F. (2021)	ASC	Cancer surgery	Adipose tissue	Yang et al. (2021)
Yan Y. (2019)	MSC	Trauma	Bone	Yan et al. (2019)
Li J. (2024)	MSC	Trauma	Cartilage	Li et al. (2024)
DeWard A. D. (2014)	Primary cell	Barrett’s esophagus	Esophagus	DeWard et al. (2014)
McCracken K. W. (2014)	iPSC	Stomach cancer	Stomach	McCracken et al. (2014)
Spence J. R. (2011)	iPSC	Inflammatory bowel disease	Intestine	Spence et al. (2011)
Takebe T. (2013)	iPSC	Cystic fibrosis	Liver	Takebe et al. (2013)
Dye B. R. (2015)	iPSC	Cystic fibrosis	Lung	Dye et al. (2015)
Boj S. F. (2015)	Primary cell	Pancreas cancer	Pancreas	Boj et al. (2016)
Fisher B. (2023)	iPSC	Heart defect	Heart	Fischer et al. (2023)
Gao D. (2014)	Primary cell	Prostate cancer	Prostate	Gao et al. (2014)
Eiraku M. (2011)	ESC	Retinitis pigmentosa	Retina	Eiraku et al. (2011)
Soucy J. (2025)	ESC	Glaucoma	Eye	Soucy et al. (2025)
Koehler K. R. (2014)	ESC	Amblyacousia	Inner ear organ	Koehler and Hashino (2014)
Hisha H. (2013)	ESC	Tongue cancer	Tongue	Hisha et al. (2013)
Millman J. R. (2016)	iPSC	Diabetes mellitus	β cell	Millman et al. (2016)
Nanduri L. S. Y. (2014)	Primary cell	Hyposalivation	Salivary gland	Nanduri et al. (2014)
Lee V. (2014)	Cell line	Burn	Skin	Lee et al. (2014)
Baltazar T. (2022)	ESC	Skin cancer	Skin	Baltazar et al. (2023)
Salas-Silva S. (2023)	iPSC	Liver disease	Liver	Salas-Silva et al. (2023)
Enbergs S. (2024)	Cell line	Trauma	Muscle	Enbergs et al. (2024)

ASC, adipose-derived stem cell; MSC, mesenchymal stem cell; iPSC, induced pluripotent stem cell; ESC, embryonic stem cell.

One of the primary advantages of 3D culture over 2D systems is the preservation of critical extracellular matrix components and cell-cell or cell-matrix interactions (Ravi et al., 2015; Gonzalez Diaz et al., 2019; Tomas-Bort et al., 2020). These interactions are essential for various cellular processes, including differentiation, proliferation, and the expression of specific phenotypic traits. By maintaining these critical elements, 3D culture systems provide researchers with a more holistic view of cellular behavior, allowing for more accurate predictions of *in vivo* responses to various stimuli and treatments.

Studies have demonstrated that 3D tissue cultures can offer novel insights into tumorigenic mechanisms that may not be apparent in conventional 2D models (Liu et al., 2019; Roberts et al., 2019; Bahcecioglu et al., 2020; Zhang et al., 2020). The three-dimensional arrangement of cells in these cultures closely mimics the architecture of tumors *in vivo*, facilitating the study of complex processes such as invasion, metastasis, and drug resistance. Additionally, 3D culture systems enable the examination of dynamic cellular behaviors, such as migration and morphogenesis, which are challenging to replicate in 2D environments (Yamada and Sixt, 2019).

Moreover, 3D culture methods offer enhanced biomarker expression, providing researchers with valuable tools for studying cellular functions and interactions (Ravi et al., 2015). By accurately recapitulating the native cellular microenvironment, these systems enable the investigation of signaling pathways and regulatory mechanisms that are crucial for understanding disease progression and treatment response (Gonzalez Diaz et al., 2019). This improved biomarker expression also enhances the sensitivity and specificity of assays, leading to more reliable experimental results.

In addition to their utility in basic research, 3D culture systems hold promise for applications in drug development and personalized medicine (Linsley et al., 2019; Brancato et al., 2020; Fernandes et al., 2020; Fisher and Rao, 2020). These systems can serve as cost-effective screening platforms for identifying potential therapeutic agents and predicting their efficacy and safety profiles. By incorporating patient-derived cells into 3D cultures, researchers can tailor treatment strategies to individual patients, improving the likelihood of successful outcomes and minimizing adverse effects.

Overall, the transition from 2D to 3D cell culture represents a paradigm shift in biomedical research, offering researchers a more physiologically relevant model system for studying cellular behavior and disease mechanisms (Gopalakrishnan, 2019). By preserving critical cellular interactions and microenvironmental cues, 3D culture methods offer a more accurate representation of *in vivo* conditions. These technologies hold the potential to revolutionize medical fields, from cancer biology to regenerative medicine, paving the way for new diagnostic and therapeutic strategies.

## Limitations

Limitations in 3D cell culture models present significant challenges both in terms of usability and standardization (Kim et al., 2020). Cultivating cells in three dimensions demands a certain level of expertise due to the necessity of forming cell aggregates, which complicates tasks such as exchanging culture medium and maintaining extracellular matrix integrity, leading to potential issues with cross-contamination during experimentation (Brancato et al., 2020). Addressing these challenges may necessitate the development of culture vessels that facilitate convenient medium exchange, thereby simplifying the experimental process (Gleave et al., 2020).

Standardization of analysis poses another significant challenge in the context of 3D cell culture models (Zhang et al., 2020). The three-dimensional nature of cell aggregates in 3D culture models introduces variability in the diffusion and penetration of these reagents (Czaplinska et al., 2019; Collins et al., 2020; Foglietta et al., 2020). Currently, common methods involve staining and measuring the size of cell aggregates, but these fail to accurately represent the activity of cells within these aggregates (Ashok et al., 2020). Efforts are underway to precisely measure cellular activity within aggregates using techniques such as tissue clearing or confocal microscopy (Ji et al., 2019; De Leon et al., 2020; Foglietta et al., 2020). However, standardization of these techniques remains elusive.

Continued research and development efforts are imperative to address these challenges and standardize the technology. Once these issues are resolved, 3D cell culture models, with their excellent biocompatibility, hold tremendous promise for applications in precision medicine, the pharmaceutical and biotechnology industries, and basic research. With further refinement and standardization, these models could revolutionize various fields by providing more physiologically relevant platforms for drug discovery, toxicity testing, disease modeling, and surgical application, ultimately leading to improved outcomes for patients (Ashraf et al., 2019).

## Conclusion

3D cell culture models offer a promising approach to studying cell behavior *in vitro*, providing a more physiologically relevant environment compared to traditional 2D cultures. Despite the challenges, advancements in 3D cell culture technology hold great potential for revolutionizing medical research and clinical practice.
